# Spa-RQ: an Image Analysis Tool to Visualise and Quantify Spatial Phenotypes Applied to Non-Small Cell Lung Cancer

**DOI:** 10.1038/s41598-019-54038-9

**Published:** 2019-11-26

**Authors:** Jie Bao, Margarita Walliander, Ferenc Kovács, Ashwini S. Nagaraj, Annabrita Hemmes, Virinder Kaur Sarhadi, Sakari Knuutila, Johan Lundin, Peter Horvath, Emmy W. Verschuren

**Affiliations:** 1 Institute for Molecular Medicine Finland (FIMM), HiLIFE, University of Helsinki, Helsinki, 00014 Finland; 2 Single-Cell Technologies Ltd, Szeged, Hungary; 3 Department of Pathology, Haartman Institute, University of Helsinki, Helsinki, Finland; 4 Department of Public Health Sciences, Karolinska Institutet, Stockholm, Sweden; 5 Synthetic and Systems Biology Unit, Hungarian Academy of Sciences, Biological Research Center, Temesvári körút 62, 6726 Szeged, Hungary

**Keywords:** Non-small-cell lung cancer, Tumour heterogeneity

## Abstract

To facilitate analysis of spatial tissue phenotypes, we created an open-source tool package named ‘Spa-RQ’ for ‘Spatial tissue analysis: image Registration & Quantification’. Spa-RQ contains software for image registration (Spa-R) and quantitative analysis of DAB staining overlap (Spa-Q). It provides an easy-to-implement workflow for serial sectioning and staining as an alternative to multiplexed techniques. To demonstrate Spa-RQ’s applicability, we analysed the spatial aspects of oncogenic KRAS-related signalling activities in non-small cell lung cancer (NSCLC). Using Spa-R in conjunction with ImageJ/Fiji, we first performed annotation-guided tumour-by-tumour phenotyping using multiple signalling markers. This analysis showed histopathology-selective activation of PI3K/AKT and MAPK signalling in *Kras* mutant murine tumours, as well as high p38MAPK stress signalling in p53 null murine NSCLC. Subsequently, Spa-RQ was applied to measure the co-activation of MAPK, AKT, and their mutual effector mTOR pathway in individual tumours. Both murine and clinical NSCLC samples could be stratified into ‘MAPK/mTOR’, ‘AKT/mTOR’, and ‘Null’ signature subclasses, suggesting mutually exclusive MAPK and AKT signalling activities. Spa-RQ thus provides a robust and easy to use tool that can be employed to identify spatially-distributed tissue phenotypes.

## Introduction

Epithelial cells are locally influenced by neighbouring stromal cells and secreted molecules, as well as tissue gradients in oxygenation and mechanical forces^1,2^. During carcinoma progression, such factors contribute to microenvironmental heterogeneity which, in conjunction with tumour cell-intrinsic genetic and molecular heterogeneity, fosters tumour evolution and compromises treatment success^3–5^. Tumour heterogeneity can be interrogated by gene-centric methods, such as multi-regional or single cell spatial sequencing, or by phenotypic assays, such as high-content *in vitro* imaging or tissue profiling using immunohistochemistry (IHC) analyses. Recent advances in multiplexed staining techniques have deepened our understanding of spatial phenotypes at tissue levels and yielded data with clinical relevance^6–8^. Although powerful in principle, practical factors limit flexible implementation of multiplexed strategies, including availability of specific antibodies of varying host species or signal unmixing accuracy. No such issues exist for the staining of serial sections using standard IHC. However, this requires image analysis software that integrates image registration, annotation, and spatial signal quantification.

NSCLC accounts for 85% of all lung cancers and constitutes a heterogeneous set of diseases with complex genomic landscapes^9,10^. Therapies targeting mutations in the EGFR and ALK proteins are available, but these mutations are only detected in a small portion of NSCLCs^11^. Personalised medicine efforts currently focus on the identification of sample-specific drug sensitivities, or synergistic combinatorial strategies. However, drug-response studies are mostly done *in vitro* using biochemical means following extrinsic modulation, despite the knowledge that oncogenic functions are dynamically and spatially regulated within tumours. Furthermore, the oncogenic signalling landscapes of tumours or cancer cell lines are typically characterised by genetic profiling^12^ or mass spectrometry-based multi-omics analysis^13^, while the spatial aspects of networked oncogenic activities in native tumour tissues remain understudied.

The PI3K/AKT and MAPK pathways are frequently activated in cancers, including *KRAS* mutant NSCLCs, and around 30% of adenocarcinomas (ACs) and 3% of squamous cell carcinomas (SCCs) contain *KRAS* mutations^14^. By using NSCLC tumours from genetically engineered mouse models (GEMMs) driven by *Kras*^*G12D*^ expression together with loss of *Lkb1* (*Kras*^*G12D*^; *Lkb1*^*fl/fl*^, hereafter called KL) or *p53* (*Kras*^*G12D*^; *p53*^*fl/fl*^, hereafter called KP), we previously demonstrated that NSCLC histopathologies exhibit differences in oncogenic signalling activities^15^. We further showed that an increased spatial overlap of phosphorylated ERK and 4EBP1, respectively marking MAPK and mTOR pathway activities, was associated with increased cytotoxic response towards co-inhibition of these pathways in murine tumour slice explants^15^. This highlights that baseline oncogenic signalling phenotypes in tumour tissues may inform on drug sensitivity. Hence, we created a Spatial tissue analysis tool package for image Registration and Quantification, called ‘Spa-RQ’, to enable image alignment and quantitative examination of spatial tissue phenotypes detected by standard histochemical 3,3’-diaminobenzidine (DAB) staining of adjacent tissue sections. This study describes the features of the Spa-RQ tool package, and demonstrates how its implementation permits dissection of the spatial oncogenic signalling profiles of native murine and human NSCLC samples.

## Results

### Introduction of the Spa-RQ software tool package

**Figure 1:**
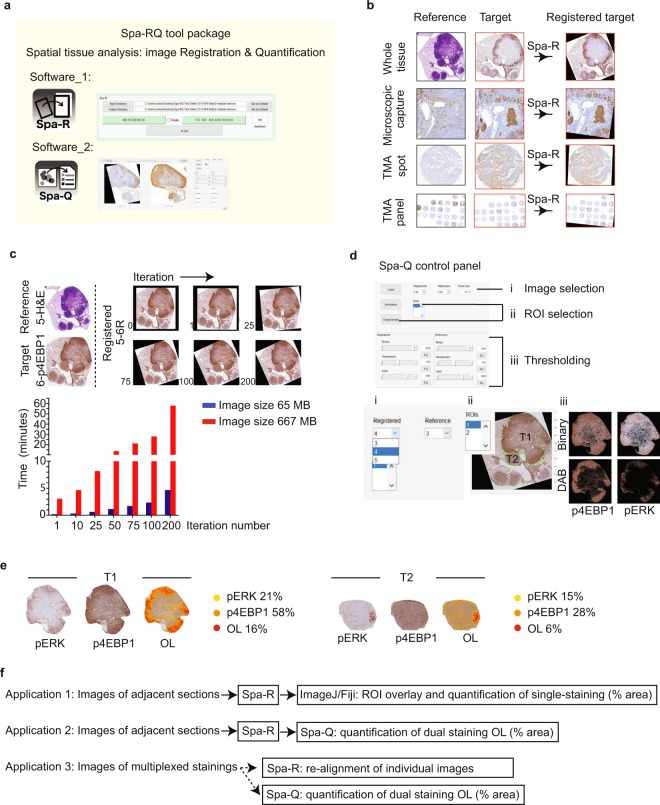
An introduction to the Spa-RQ software tool package. (**a**) Schematic overview of Spa-RQ, which includes software for image registration (Spa-R) and spatial quantification (Spa-Q). (**b**) Examples of image sets that can be registered by Spa-R. (**c**) Upper image panel illustrates the alignment outcomes following Spa-R implementation with different iteration numbers. The registration quality increases with the iteration number, which can be set by users. The lower bar plot shows how the time required for Spa-R registration increases with image size and iteration number. An iteration rate of 100 is recommended to register images on neighbouring 4 µm tissue sections. (**d**) Overview of the control panel of Spa-Q. A detailed User Manual can be found in the software folder at the official repository. (**e**) Examples of outputs generated by Spa-Q. For each ROI, a coloured overlay mask is provided in conjunction with an Excel file listing image quantification data. (**f**) Overview of various image modalities to which Spa-RQ can be applied.

The Spa-RQ tool package was designed for spatial phenotyping on stained serial sections, as an easy-to-implement alternative to multiplexed strategies. It contains an image registration program named ‘Spa-R’, and a DAB quantification program named ‘Spa-Q’ (Fig. 1a). Spa-RQ is open-source, compatible with both Windows and Mac systems. In comparison to other open-source methods/software that operate on whole slide scans of serial sections^16–18^, it uniquely combines multiple image processing functions with a capacity to perform spatial analysis in selected regions-of-interest (ROIs).

Spa-R iteratively applies a similarity transformation method (the MATLAB^®^ function imregtform) for image registration. It can be used to either register pairs or batches of input images in a variety of formats, including TIFF, JPEG or PNG, ranging from MB to GB in size. Image registration using Spa-R can be applied to a wide range of image captures, and generates newly registered uncompressed TIFF images as output (Fig. 1b). The iteration rate of the registration process can be manually adjusted, dependent on precision vs. processing speed considerations for a registration case (Fig. 1c). While selective additional parameters can be tested for registration optimization using Spa-R_optimizer (Supplementary Fig. S1a,b), Spa-R’s entire codebase is provided to enable computational optimization on registration tasks. A comparison of parameter optimization relative to the default settings, used for the registration of large >500 MB images, shows that the number of iterations is the major factor determining registration quality relative to registration speed (Supplementary Fig. S1d), while other factors have either less (PyramidLevels and InitialRadius; Supplementary Fig. S1c,e) or no effect (Epsilon and GrowthFactor; Supplementary Fig. S1f,g). Spa-R can be installed on multiple computers, and can thus be run in parallel with Spa-Q for optimal efficiency.

The Spa-Q image analysis program measures the areas (%) of two individual DAB signals and the area (%) of their overlap (OL). It is easy to operate and suitable for researchers with limited image analysis expertise. The control panel of Spa-Q includes multiple image processing modules, specifically (i) image selection, (ii) ROI creation/selection, and (iii) thresholding-based segmentation of tissue areas (binary thresholding), hematoxylin (segmentation of nuclei) and DAB-stained areas (Fig. 1d). Following image processing, the results folder is automatedly loaded with segmented images and an Excel sheet that lists the raw signal quantification results (percentages of individual DAB-stained areas, and area of OL per ROI). In addition, a pseudo-coloured mask depicting the two individual stainings in yellow and orange, and their OL area in red, is generated (Fig. 1e).

The next sections describe two proof-of-concept applications of the Spa-RQ tool package. In the first, Spa-R was used in conjunction with ImageJ/Fiji^19^ to perform annotation-guided quantification of multiple stainings on serial tissue sections (Application 1 in Fig. 1f). In the second application, Spa-RQ was used to quantify the spatial co-expression of two signalling markers, detected by staining on neighbouring tissue sections (Application 2 in Fig. 1f). Another possible application, not introduced here, is for re-alignment or quantification of multiplexed images (Application 3 in Fig. 1f). Importantly, Spa-RQ’s source code is freely available, and can be modified to match the user’s needs, for example to enable the quantitative analysis of fluorescent signals.

### Using Spa-R for NSCLC histopathology-selective lesion profiling

**Figure 2:**
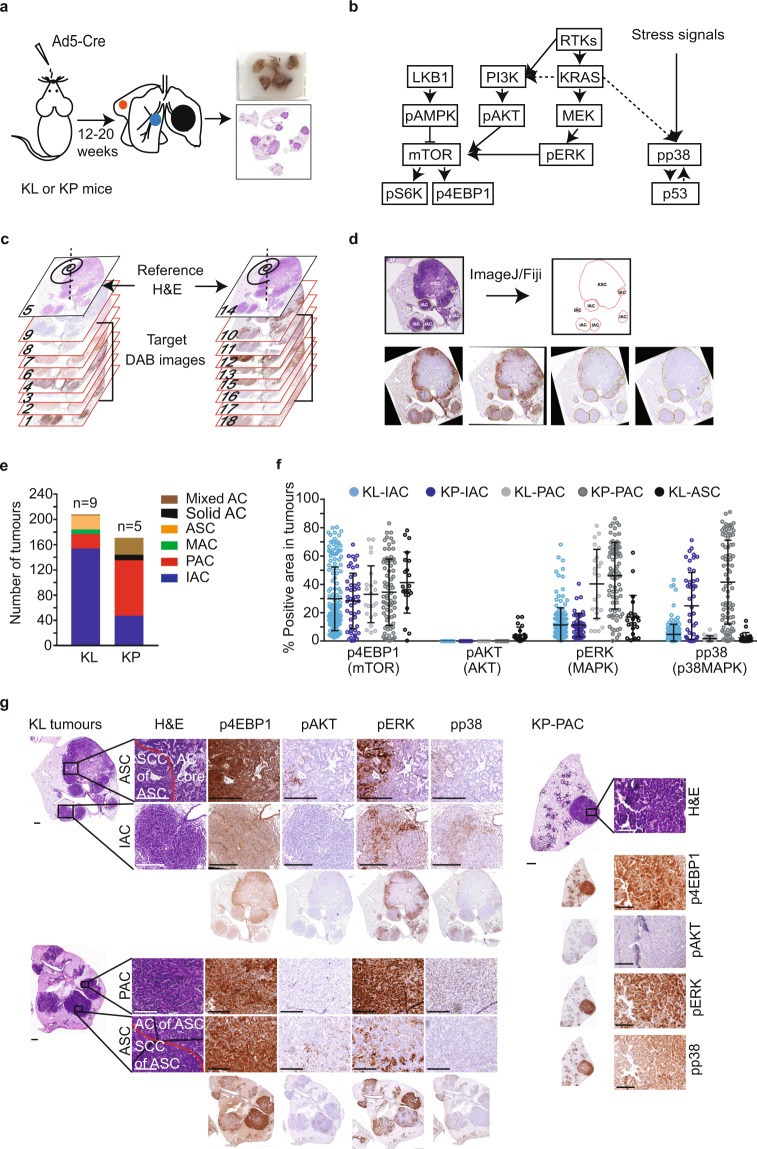
Implementation of Spa-R to analyse oncogenic signalling activities in murine NSCLC histopathologies. (**a**) Schematic overview depicting FFPE lung tumour tissue block preparation from *Kras*^*G12D*^; *Lkb1*^*fl/fl*^ (KL) or *Kras*^*G12D*^; *p53*^*fl/fl*^ (KP) GEMMs. Multiple tumours arise in the lung lobes of an individual GEMM. (**b**) Diagram illustrating oncogenic signalling pathways regulated by KRAS and LKB1 or p53. Solid arrows indicate direct regulation. (**c**) Schematic overview showing the order of H&E- and DAB-stained tissues sections for image registration. (**d**) Diagram showing an example image analysis workflow using Spa-R for registration and ImageJ/Fiji for annotation and quantification. The former is used to align DAB images to H&E images, the latter is used to generate annotations and perform ROI-guided quantification of DAB signal on each of the registered images. Red outlines on DAB images are original overlays from H&E-based annotations; green outlines are adjusted overlays due to the distance between tissue sections stained with different antibodies. (**e**) Graphical representation of the numbers of NSCLC lesions of different histopathologies in 9 KL and 5 KP GEMMs. In mixed ACs (adenocarcinomas) multiple histological features are found in one tumour. (**f**) Scatterplot showing areas (%) of p4EBP1 (marking mTOR activation), pAKT (marking AKT activation), pERK (marking MAPK activation) and pp38 (marking p38 activation) in individual adenosquamous carcinomas (ASCs), papillary adenocarcinomas (PACs), or invasive adenocarcinomas (IACs). Statistical analysis: one-way ANOVA multiple comparison (Kruskal-Wallis test, nonparametric test) with uncorrected Dunn’s test. (**g**) Representative images illustrating ASC, PAC, and IAC histological features (H&E staining), as well as the stainings of p4EBP1, pAKT, pERK, and pp38 in a histopathology-specific manner. Scale bars, 500 µm for KL upper panel (entire) images and KL lower panel H&E image and KP H&E image; 100 µm for the others.

The expression of oncogenic *Kras* together with loss of *Lkb1* or *p53* in murine lung progenitor cells gives rise to a spectrum of NSCLC lesions of different histopathologies^20,21^. These mice develop multiple tumours in the lungs, each marked by distinguishable histological features (Fig. 2a). We set out to use Spa-R to quantify NSCLC histopathology-selective oncogenic signalling activities, with primary focus on the PI3K/AKT, MAPK, and mTOR pathways, as well as the p38MAPK stress-sensing pathway (Fig. 2b). To achieve this, Spa-R was used to register DAB-stained images to their closest H&E reference (Fig. 2c). Registered DAB images were subsequently overlaid with the ROI mask generated by annotating the H&E references using ImageJ/Fiji, to permit quantification of DAB signal per ROI with ImageJ/Fiji (Fig. 2d).

Lesions of the three most common histopathology types in KL and KP GEMMs, namely adenosquamous carcinoma (ASC), confined to KL mice, as well as papillary adenocarcinoma (PAC) and invasive adenocarcinoma (IAC) (Fig. 2e) were subjected to quantification of p4EBP1 (marking mTOR), pAKT (marking AKT), pERK (marking MAPK), and pp38 (marking p38MAPK) expression (Fig. 2f, Supplementary Fig. S2a). As reported^15^, phosphorylation of AKT was detected in ASCs but absent in PACs and IACs (P value < 0.0001). Furthermore, pERK expression was significantly higher in PACs than in IACs, suggesting differential MAPK regulatory phenotypes in these two AC subtypes (KL-PAC vs KL-IAC, individual tumours P value < 0.0001, grouped by animal P value = 0.001; KP-PAC vs KP-IAC, individual tumours P value < 0.0001, grouped by animals P value = 0.0135). MAPK signalling was activated more widely in PACs than ASCs (individual KL-PAC vs ASC P value = 0.0024, grouped by animals P value = 0.0257) (Fig. 2f,g, Supplementary Fig. S2a). p4EBP1 expression, on the other hand, was variable from lesion to lesion, showing no significant differences between the three histopathologies (Fig. 2f,g). Thus, NSCLC subtype-selective oncogenic signalling signatures were observed, with ASCs marked as ‘AKT^positive^/MAPK^low-medium^’, PACs as ‘AKT^negative^/MAPK^medium-high^’ and IACs as ‘AKT^negative^/MAPK^low-medium^’. Interestingly, p38MAPK phosphorylation was found to be genotype-selective, showing pathway activation specifically in KP tumours (Fig. 2f,g). Implementation of Spa-R as an image pre-processing step is thus shown to achieve ROI-guided staining quantification, and identifies oncogenic signalling phenotypes characteristic for ASC, PAC, and IAC NSCLC histopathology types.

### Using Spa-RQ to visualise and quantify the spatial features of phosphoprotein signals in murine NSCLC tumours

**Figure 3:**
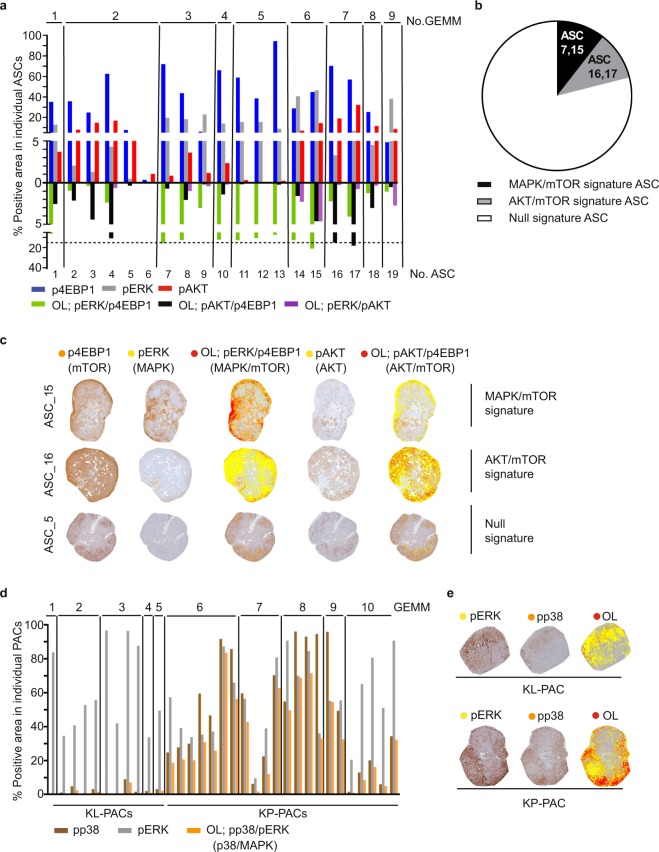
Application of Spa-RQ in phenotyping oncogenic signalling network signatures. (**a**) Bar plot showing the % area of p4EBP1, pERK, pAKT, as well as pERK/p4EBP1 (marking MAPK and mTOR co-activation), pAKT/p4EBP1 (marking AKT and mTOR co-activation) and pERK/pAKT (marking MAPK and AKT co-activation) overlap in individual ASCs from 9 KL mice. Animals no.1–5 were used in the analysis in Fig. 2e–g. Animals no. 6–9 are included only in the analysis in Fig. 3. The tumour samples were processed for pAKT, pERK, and p4EBP1 spatial analysis. The staining orders were mentioned in detail in Methods section. The dashed black line indicates a 15% OL level. (**b**) Pie chart showing the distribution of analysed ASCs in the following three signature subclasses: 1. ‘MAPK/mTOR’ (OL; pERK/p4EBP1 > 15%), 2. ‘AKT/mTOR’ (OL; pAKT/p4EBP1 > 15%) and 3. ‘Null’ signature. (**c**) Examples of ASC tumours showing either a ‘MAPK/mTOR’, ‘AKT/mTOR’ or ‘Null’ signature. (**d**) Bar plot showing the pp38 and pERK quantification as well as OL of pERK/pp38 (marking MAPK and p38 co-activation) by Spa-RQ in individual PACs grouped per animal. (**e**) Representative landscape images illustrating the spatial distribution of pERK and pp38 in one KL-PAC and one KP-PAC, generated by Spa-RQ.

Our previous findings linked the extent of spatial co-activation of mTOR and MAPK pathways to their combinatorial inhibitory response in slice explants^15^. We hence wished to demonstrate the use of Spa-RQ in measuring the spatial co-activation of signalling pathways to dissect histopathology-selective phenotypes. Spa-RQ was applied to measure the expression of pERK, pAKT, p4EBP1, and their mutual areas of OL (marking MAPK-mTOR co-activation and AKT-mTOR co-activation respectively) in individual murine ASC lesions (Fig. 3a). Using a cut-off of >15% OL of pERK and p4EBP expression, as was used in^15^, Spa-RQ analysis showed 2 of 19 ASCs to have a ‘MAPK/mTOR’ signature (ASC_OL; pERK/p4EBP1 > 15%). This is lower than found before (4 out of 9 ASCs in^15^), indicating that quantification of manually-drawn stained area masks, as was done previously, can overestimate spatial biomarker quantification compared with intensity-based signal segmentation as done here. Additionally, 2 of 19 ASCs were shown to have an ‘AKT/mTOR’ signature (ASC_OL; pAKT/p4EBP1 > 15%). The remaining (15/19) ASCs were classified as having a ‘Null’ signature (Fig. 3b). Lesions never carried both ‘MAPK/mTOR’ and ‘AKT/mTOR’ signatures (Fig. 3b), indicating mutual exclusivity, consistent with the low OL of pERK and pAKT expression in ASC tumours (Fig. 3c). ‘MAPK/mTOR’ and ‘Null’ signature tumours co-existed in animals #3 and 6, indicating inter-tumour signature heterogeneity. We similarly analysed the spatial co-activation of the MAPK and mTOR pathways in AKT^negative^/MAPK^medium-high^ papillary adenocarcinoma (PAC) tumours, which showed that PACs either classified as ‘MAPK/mTOR’ (PAC_OL; pERK/p4EBP1 > 15%) or as ‘Null’ signature tumours, again indicating inter-tumour signalling heterogeneity within this histopathology group (Supplementary Fig. S2b,c). Taken together, quantitative analysis of signalling OL with Spa-RQ revealed that murine NSCLC lesions subclassify based on the levels of spatial MAPK/mTOR or AKT/mTOR co-activation.

Finally, we applied Spa-RQ to investigate if there was any evidence of signalling pathway co-activation in KP-PACs which showed selectively high p38MAPK activity. Interestingly, high p38 phosphorylation was detected specifically in regions showing concomitant MAPK activation (Fig. 3d,e). Indeed, even the small areas showing pp38 positivity in KL-PACs were in regions showing high MAPK activity, tentatively suggesting dependency on MAPK activation to elicit detectable p38MAPK pathway activation in murine PACs.

### Spa-RQ reveals spatial oncogenic phenotypes in clinical NSCLC samples

To investigate to what extent the histopathology-selective spatial signalling phenotypes detected in murine samples were representative of human NSCLC, we used Spa-RQ to analyse a set of clinical samples. We first visually evaluated the positivity of MAPK, AKT, mTOR, and p38MAPK activation in TMA panels of clinical ASCs and PACs (Supplementary Fig. S3a). As reported in^15^, AKT activation was seen uniquely in clinical ASCs (5/13), but absent in PACs (0/25). However, unlike GEMM tumours, where the % area range of pERK expression was clearly distinguishable between ASCs (pERK^low-medium^) and PACs (pERK^medium-high^), we did not find such signatures in human samples. Instead pERK^medium-high^ ASCs and pERK^low-medium^ PACs were the most common phenotypes in clinical NSCLCs (Supplementary Fig. S3b). Moreover, pp38 positivity was observed in more clinical ASCs (61%) than in PACs (40%), differentiating from the selectively high pp38 expression in murine KP-PACs (Fig. 2f,g, Supplementary Fig. S3a,b). Therefore, histopathology-selective PI3K/AKT activation, but not MAPK or p38MAPK activation, was conserved between murine and clinical NSCLC tumours.

**Figure 4:**
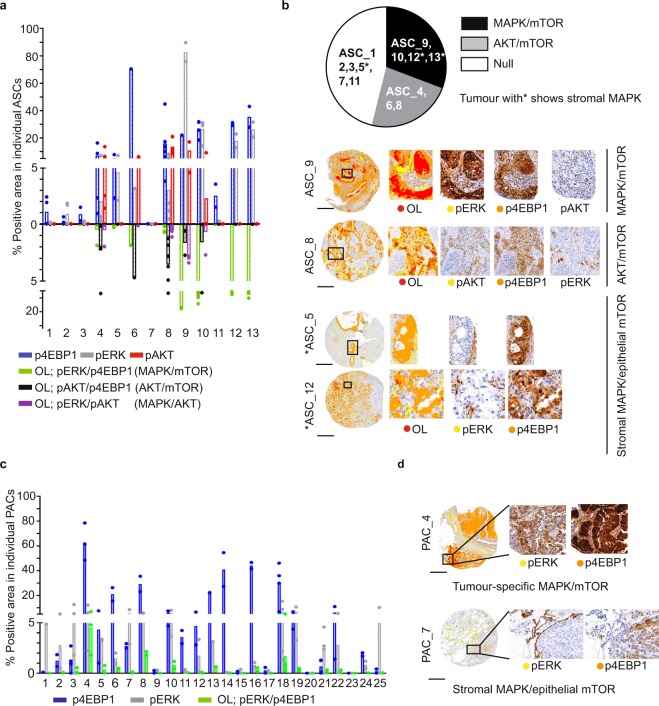
Spa-RQ reveals spatial oncogenic phenotypes in NSCLC TMA samples. (**a**) Scatterplot depicting the areas (%) of p4EBP1, pERK, pAKT, as well as pERK/p4EBP1, pAKT/p4EBP1 and pERK/pAKT overlap in 13 individual clinical ASCs. Each dot represents a TMA spot. Human NSCLC TMA blocks contains 2–3 replicate cores, representing two spatially distinct tissue regions, and 2–6 TMA spots per ASC tumour were analysed. Bars indicate the average values among the spots within one ASC. (**b**) Pie chart illustrating the classification of ASCs according to mTOR/MAPK/AKT spatial phenotypes. Representative images illustrating the classes shown in the pie chart, with exception of the ‘Null’ signature. Tumours labelled with an asterisk* show stromal MAPK and tumour-specific mTOR signalling. On landscape mask, OL areas are marked with red colour, yellow and orange each present tumour area stained with an individual marker. Scale bar, 500 µm. (**c**) Scatterplot showing the expression of p4EBP1 and pERK, as well as their OL in 25 clinical PAC samples. Each dot represents a TMA spot, and 2–4 spots representing spatially distinct tissue regions per PAC sample were included in the analyses. Bars indicating the average value of spots for individual PAC. (**d**) Representative images of PAC TMA samples showing a tumour-specific ‘MAPK/mTOR’ signature or stromal MAPK/epithelial tumour mTOR signature. Scale bar, 500 µm.

Noting that pERK, pAKT, p4EBP1, and pp38 were spatially expressed also in clinical NSCLC samples (Supplementary Fig. S3b), we next wished to explore if spatial measurements with Spa-RQ could identify signalling co-activation signatures selective for clinical NSCLC subtypes. We therefore applied Spa-RQ to investigate whether the classification of tumours based on MAPK/mTOR (OL; pERK/p4EBP1) or AKT/mTOR (OL; pAKT/p4EBP1) co-activation signatures translates to human samples. First, the individual levels and OL expression of pERK, p4EBP1, and pAKT were measured on a spot-by-spot manner for each ASC (Fig. 4a). Differently from murine tumour analyses, classifications were made according to the dominant phenotypes, as intra-tumour stromal infiltrates and overall decreased areas of staining obviated use of a 15% OL cut-off value. We found that human ASCs similarly stratified into the three subclasses identified by murine tumour phenotyping, namely: (i) ‘MAPK/mTOR’ signature tumours (ASC_9, 10, 12, 13; 4/13); (ii) ‘AKT/mTOR’ signature tumours (ASC_4, 6, 8; 3/13); and (iii) ‘Null’ signature tumours (ASC_1, 2, 3, 5, 7, 11; 6/13) (Fig. 4b). Variations were measured in the duplicate spots sampled from the same donor blocks, indicating intra-tumour signalling heterogeneity. We similarly performed spatial analysis on TMA sections representing 25 clinical PAC samples (Fig. 4c), and confirmed that both ‘MAPK/mTOR’ signature (PAC_4,8,18) and ‘Null’ signature tumours were detected in clinical PACs. Importantly, of the samples for which the *KRAS* mutation status is known, no correlation between *KRAS* mutation and spatial signalling signature was detected (Supplementary Fig. S4a,b). The spatial signalling pathway co-activation signatures of murine tumours were also identified in the clinical samples.

Based on the quantitative data as well as the landscape masks provided by Spa-RQ analysis, we observed a phenotype of stromal MAPK signature in conjunction with tumour-specific mTOR signalling, in both ‘MAPK/mTOR’ (ASC_12, 13) and ‘Null’ (ASC_5) signature ASCs (Fig. 4a,b). Furthermore, a heterotypic tumour-stromal distribution of MAPK and mTOR pathways was also observed in ‘Null’ signature PAC tumours (Fig. 4c,d). This indicates increased signalling heterogeneity in clinical tumours, associated with the heterotypic stromal infiltration characteristic of clinical tumours.

Finally, we also explored the spatial activation of p38MAPK signalling in clinical samples, and asked if p38 phosphorylation was detected in conjunction with high pERK expression, as was the case in murine PACs. Of the eight pp38^positive^ ASCs, four contained a ‘MAPK/mTOR’ signature, and only one of these (ASC_9, pERK^high^) showed spatial co-activation of MAPK and p38MAPK signalling. In addition, three pp38^positive^ ASCs containing an ‘AKT/mTOR’ signature (ASC_4, 6, 8) showed pp38 staining in pAKT positive areas (Supplementary Fig. S3c). In clinical PACs, the co-activation of MAPK and p38MAPK was observed in only one out of ten pp38^positive^ tumours (PAC_4, ‘MAPK/mTOR’ signature) (Supplementary Fig. S3d). Thus, the spatial p38MAPK and MAPK co-activation phenotype of clinical samples was more heterogeneous than in murine samples. In conclusion, implementation of Spa-RQ can classify both murine and clinical NSCLCs based on distinct signalling co-activation signatures, and reveals that histopathology-selective PI3K/AKT activity in ASC tumours is conserved between species. However, the more pronounced molecular and heterotypic stromal heterogeneity of human NSCLCs adds complexity to straightforward phenotype-based disease stratification.

## Discussion

We introduce a software package named Spa-RQ to visualise and quantify the spatial features of markers detected by standard IHC on serial tissue sections. This package is easy to operate and visualises the spatial data as an attractive landscape. Spa-RQ consists of the Spa-R program for image registration and the Spa-Q program for spatial image analysis in multiple ROIs on IHC images. Image registration is a much anticipated method development field, and sophisticated algorithms are required for lower resolution medical images or for motion correction^22,23^. However, the histological images in our study are applied to serial sections of tissues with near-identical architecture, and stained with standardised protocols. A similarity transformation with mutual information registration method therefore have satisfied all our tests and is used in Spa-R. This image pre-processing step enables spatial analysis, e.g. using Spa-Q or ImageJ/Fiji, at the tissue level. Both of these apply simple signal intensity-based quantification methods, necessitating an inter-observer study to set thresholds. Implementation of Spa-RQ for the phenotypic profiling on any set of DAB-stained slides can help discover native tissue phenotypes, and generate hypotheses for further investigation. Spa-RQ is open-source software and can be further modified based on the user’s needs, for example: to quantify spatial co-expression of more than two markers; or to add segmentation modules for spatial analysis at single-cell resolution.

As proof-of-concept demonstrations of Spa-RQ, we first combined use of Spa-R and ImageJ/Fiji to analyse the signalling activities in NSCLC lesions of different histopathologies. This corroborated our previous identification^15^ of histopathology-selective signalling signatures, reflecting the diverse roles of signalling networks in oncogenic transformation. Specifically, we found that *Kras* mutant murine ASCs, PACs, and IACs were classified as AKT^positive^/MAPK^low-medium^, AKT^negative^/MAPK^medium-high^, and AKT^negative^/MAPK^low-medium^, respectively, and that the PAC and IAC signatures were independent of the KL and KP driver mutations. Similar to the murine sample data, clinical PACs also showed absence of AKT signalling, suggesting that signalling mechanisms driving histopathology-selective lesion progression may in part be conserved across species. Functional studies with lung cancer cell lines have similarly demonstrated that MAPK versus PI3K/mTOR dependency can stratify KRAS-driven cancers^24^, and an interesting outstanding question remains how this may relate to histopathology-selective pathway dependencies.

In the second proof-of-concept application, we applied Spa-RQ as a spatial analysis tool to demonstrate how oncogenic signalling pathway co-activation can be examined in individual tumours. The MAPK and PI3K/AKT pathways have multiple downstream effectors, including mTOR pathway activation, which have major roles in tumourigenesis, disease prognosis, and therapeutic response^25,26^. ASC tumours showed heterogeneity in MAPK, AKT, and mTOR pathway activities, and were stratified into three mutually exclusive subclasses, namely ‘MAPK/mTOR’, ‘AKT/mTOR’, and ‘Null’. Minimal co-activation of the MAPK and AKT pathways was observed, which is consistent with the known negative feedback regulation of MAPK activity by AKT^27^. Similarly, NSCLC PACs were divided into ‘MAPK/mTOR’ and ‘Null’ tumours. Importantly, the same tumour subclasses were observed in human samples, indicating cross-species conservation. In some clinical samples, clear stromal MAPK activity could be detected, in accordance with the increased stromal component in human disease compared to murine tumours. The detection of signalling heterogeneity in tumours carrying identical driver mutations matches a previous report demonstrating PI3K/MAPK heterogeneity in PIK3CA mutant breast cancer, a rare example of a study applying multiplexed techniques to investigate phosphoprotein networks^28^. An interesting question for future mechanistic exploration is whether such spatial phenotyping of *in situ* signalling heterogeneities can further refine the predictive stratification of tumours linked to drug response.

Contrary to the histopathology-selective MAPK and AKT activation patterns, p38MAPK activation was found to be associated with p53 loss-of-function in murine NSCLC. This is potentially explained by increased ATM/ATR-mediated phosphorylation of p38 in p53 null cells, known to promote cell survival following DNA damage^29^. Corroborating this, a more recent study showed that activation of p38MAPK has an active role in DNA repair by phosphorylation of CtIP^25^, together suggesting that the increased p38MAPK signalling relates to compromised DNA integrity of p53 null tumours. Interestingly, we found that p38 phosphorylation was particularly pronounced in murine tumour regions with high MAPK activity, implying context-dependent co-activation of p38MAPK and MAPK pathways following DNA damage or other stress mechanisms. However, in clinical samples, p38MAPK activation was not clearly associated with increased MAPK activity, consistent with a more intricate molecular evolutionary diversity of clinical samples, compromising simple correlations.

To conclude, Spa-RQ is a simple and robust image analysis tool that, when combined with a serial section staining strategy, facilitates the identification of spatial tissue phenotypes. Application of the software in the analysis of oncogenic signalling can help to dissect inter- and intra-tumour phenotypic heterogeneity. Once linked to drug response studies, such spatial phenotyping approaches may have the potential to expose biomarkers of drug sensitivity.

## Methods

### Human and murine tumour tissues

Breeding of KL and KP mice, and lung tumour initiation, were performed as described^21^. Animal handling and studies were performed according to the guidelines from Finnish National Board of Animal Experimentation, and they were approved by the Experimental Animal Committee of the University of Helsinki and the State Provincial Office of Southern Finland (ESAVI/857/04.10.07/2013). Formalin fixed paraffin embedded (FFPE) samples of human NSCLC ASC and PAC lesions were collected from the Helsinki Biobank’s pathology sample archive (Helsinki Biobank project number HBP2016002). A TMA was constructed from the FFPE samples. Surgically resected tumour specimens were received from NSCLC patients, with informed consent, at the Hospital District of Helsinki and Uusimaa (HUS), as approved by the ethical board of the Joint Authority for the HUS, Finland (Dnro: 85/13/03/00/15). Experimental procedures on human tumour tissues were performed in accordance with the guidelines and regulations in the acquired ethical permission. Detailed information and raw data from the murine and human NSCLC sample analyses is provided in the Supplementary Table S1.

### Tissue processing, sectioning and staining order

Murine lung tumours were processed as described^15^. FFPE tissue blocks containing whole murine lung or clinical NSCLC TMA samples were cut as 4 µm thick sections. Two sequential sections were placed on one glass slide (SUPERFROST ^®^ PLUS, Thermo scientific, Ref J1800AMNZ), and labelled to mark the sectioning order. Adjacent tissue sections were each stained with one antibody from a defined series of sequential antibodies. For murine samples in Figs. 2e,g and 3a GEMM No. 1–5, stainings and tissue sections were as follows: 1-p63, 2-NKX2.1, 5-HE, 6-p4EBP1, 9-pAKT, 10-pERK, 11-pp38 and 14-HE. Thus, p4EBP1- and pAKT-stained sections were 12 µm apart, and p4EBP1- and pERK-stained sections were 16 µm apart; H&E-stained sections were 4–16 µm apart from various DAB-stained sections; pAKT-, pERK-, and pp38-stained sections were consecutive. For the additional GEMM tumour samples included in Fig. 3a GEMM No.6–9, pAKT, p4EBP1 and pERK were stained on consecutive tissue sections. For the TMA analyses, the staining order was: 1-pERK, 2-pp38, 3-pAKT and 4-p4EBP1.

### Immunohistochemistry (IHC) and image acquisition

Standard IHC was performed as described^15^. To achieve consistency in hematoxylin and DAB signals, IHC was simultaneously performed on all samples in the following groups: 1. murine samples used in Figs. 2 and 3 (No. 1–5 in Fig. 3a); 2. samples from animals 6–9; 3. TMA panels of clinical samples.

The following antibodies were used for IHC: rabbit anti-TTF1 (NKX2.1) (Abcam 133638; 1:2000), rabbit anti-p63 (Abcam ab124762; 1:10000), rabbit anti-E-cadherin (Cell Signalling Technology CST 3195; 1:400), rabbit anti-phospho-AKT for murine samples (Cell Signalling Technology CST4058; 1:400), rabbit anti-phospho-AKT for clinical samples (Cell Signalling Technology CST4060; 1:300), rabbit anti-p44/22 (Erk1/2) (Cell Signalling Technology CST4370; 1:1000 for murine samples; 1:500 for TMA samples), rabbit anti-p4EBP1 (CST2855, Cell Signalling Technology; 1:1000), rabbit anti-pp38 (Cell Signalling Technology CST4511; 1:200). For all the antibodies, antigen retrieval was done using 10 mM sodium citrate pH 6.0. All antibodies except p4EBP1 were incubated 1.5 h at room temperature; p4EBP1 antibody was incubated overnight at 4 °C. BrightVision poly-horseradish peroxidase goat anti-rabbit IgG (Immunologic, Duiven, The Netherlands) was incubated at room temperature for half an hour and Bright-DAB Substrate Kit (Immunologic, Duiven, The Netherlands) were used for detection.

Stained murine lung sections were scanned with a Cytation™ 5 Cell Imaging Multi-Mode Reader using a 10x objective, and stored as TIFF images. Whole slide scans of stained clinical TMA sections were acquired by a Pannoramic 250 3DHISTECH digital slide scanner using a 20x objective. TIFF images of individual TMA spots were exported using the Pannoramic Viewer (3DHISTECH Ltd) at a magnification of 1:2. Histopathology classification was performed as done previously^15,21^, in conjunction with an expert pathologist.

### Software development

Spa-RQ was written in MATLAB^®^ (The MathWorks, Inc. USA). It is freely distributed at https://bitbucket.org/MagoBitbucket/spa-rq-tools as an open-source tool, where users can read the general description and license; they can also go to ‘Source’ to view or use the source code, or ‘Downloads’ to access Mac or PC versions of Spa-RQ, where a User Manual is included. For the registration algorithm, the MATLAB^®^ function imregtform was used (https://se.mathworks.com/help/images/ref/imregtform.html). Spa-R iteratively applies a similarity transformation method for image registration. This uses a multimodal image input to compute an initialization for the registration metric as well as an optimizer configuration. This metric is defined by the maximization of the mutual information between the reference image and the image(s) to be aligned. The optimisation runs to a maximum number of iterations, or until it converges. While default parameters are used in Spa-R (e.g. InitialRadius = 0.0018, PyramidLevels = 3, Epsilon = 1.5e-06, and GrowthFactor = 1.05), we created Spa-R_optimizer to allows users to manually adjust all five parameters (https://bitbucket.org/MagoBitbucket/spa-rq-tools). Spa-Q performs colour decomposition with two vectors, one for Hematoxylin staining and the other for DAB. Colour decomposition matrix [0.650 0.704 0.286] is used for Hematoxylin, and [0.072 0.954 0.283] for DAB. This matrix was obtained via colour deconvolution analysis using the ImageJ/Fiji plugin^30^.

### ROI-specific image analysis with ImageJ/Fiji

ImageJ/Fiji (https://fiji.sc) was used to generate annotations: First, regions-of-interest (ROIs), in this case individual lesions on murine lung lobes, were each outlined manually with the polygon tool on H&E images. Secondly, polygons were added to the ROI manager (ImageJ/Fiji- > Analyse- > Tools > ROI manager), and named according to their histopathology classification. Third, the ROI manager for each sample was saved as a Zip file in the sample image folder. Finally, to apply the ROI library on DAB images in the image set of one tissue block, both the registered images and the ROI manager were opened in ImageJ/Fiji, and ROIs overlaid on images by selecting the “show all” function in the ROI manager. If necessary, polygons were adjusted on new images, and adjusted ROI managers saved.

For image analyses using ImageJ/Fiji, the ROI managers were first applied to each image, followed by colour deconvolution to obtain pure DAB masks (ImageJ/Fiji- > Color- > Color Deconvolution- > Vector: H DAB). The threshold for each staining was set as the average threshold values of analyses performed by three independent immunohistochemical experts, on multiple ROIs. Identical thresholds for each individual staining were applied to all samples (Supplementary Table S1). Identical thresholds for each individual staining were applied to all samples (Supplementary Table S1). To measure the tissue area, the original images were converted to 8-bit grayscale images (ImageJ/Fiji- > Image- > 8-bit), and binary thresholds were then applied. The % positivity of each staining in each tumour (regions-of-interest) were quantified as percentages of DAB-stained areas relative to the whole tumour area. All raw results and parameters were collected in Excel sheets and can be found in Supplementary Table S1.

### Spatial quantification with Spa-RQ

A step-by-step installation and user manual for Spa-RQ is included in the Spa-RQ-Windows-v1.0.zip and Spa-RQ-MACOS-v1.0.zip (https://bitbucket.org/MagoBitbucket/spa-rq-tools/downloads). The threshold for each staining was set as the average threshold values of analyses performed by three independent experts on multiple ROIs. For the analysis of murine samples, thresholding parameters were set manually to match those used in ImageJ/Fiji (Supplementary Table S1). Spa-RQ generates an Excel sheet that provides DAB and binary thresholds, and raw results data. A landscape of staining overlap, and the segmented images, are automatically saved in the output folder.

### Statistical analysis

Data visualisation and statistical analyses were performed with GraphPad Prism7 (GraphPad Software, Inc. San Diego, USA). For statistical comparisons, a one-way ANOVA multiple comparison (Kruskal-Wallis test, nonparametric test) with uncorrected Dunn’s test was used. P values < 0.05 were considered significant. Data are presented as individual dots with the mean indicated by bars, or as mean ± SD.

## Supplementary information

Table 1

Supplementary figures and legends

## Data Availability

The datasets analysed during the current study are available as Supplementary Table S1. Spa-RQ-V1.0 is freely distributed at https://bitbucket.org/MagoBitbucket/spa-rq-tools as an open-source tool. It is written in MATLAB^®^ R2016a with an academic license. The source code, standalone versions, User Manual, and additional notes are available at the official repository. Further developing the source code requires MATLAB^®^ license. However, Windows and Macintosh standalone compiled versions— that do not require license— are also available online.
