# Supplementary material for: Spa-RQ: an Image Analysis Tool to Visualise and Quantify Spatial Phenotypes Applied to Non-Small Cell Lung Cancer
Source: Sci Rep. 2019 Nov 26;9:17613. doi: 10.1038/s41598-019-54038-9 (PMC6879493; doi:10.1038/s41598-019-54038-9)
Supplement: Supplementary file 2 — Supplementary figures and legends [file 41598_2019_54038_MOESM2_ESM.pdf]

Spa-RQ: an Image Analysis Tool to Visualise and Quantify Spatial Phenotypes Applied to Non-Small Cell Lung Cancer

Jie Bao[1], Margarita Walliander[1], Ferenc Kovács[2], Ashwini S. Nagaraj[1], Annabrita Hemmes[1], Virinder Kaur Sarhadi[3], Sakari Knuutila[3], Johan Lundin[1,4], Peter Horvath[1,5] and Emmy W. Verschuren[1,*]

[1] Institute for Molecular Medicine Finland (FIMM), HiLIFE, University of Helsinki, Helsinki 00014, Finland

[2] Single-Cell Technologies Ltd, Szeged, Hungary

[3] Department of Pathology, Haartman Institute, University of Helsinki, Helsinki, Finland

[4] Department of Public Health Sciences, Karolinska Institutet, Stockholm, Sweden

[5] Synthetic and Systems Biology Unit, Hungarian Academy of Sciences, Biological Research Center, Temesvári körút 62, 6726 Szeged, Hungary

[*]Correspondence: emmy.verschuren@helsinki.fi

Figure S1. Depiction of Spa-R_optimizer configuration. (a) Data list showing the default optimizer settings of Spa-R generated by multimodal image registration with MATLAB function imregtform. (b) Display of Spa-R_optimizer's user-friendly interface, which enables adjustment on five optimizer parameters. (c) Image set comparing the registration quality at different PyramidLevels (L). Left: a pair of reference and target images (size>500MB). Right upper lane: the images registered at PyramidLevels 1-5 (L1-5). Right lower lane: zoom-in overlapped images to compare registration quality, with reference image at back (at 100% opacity) and registered image in front (at 70% opacity). Asterisks indicate low (*), medium (**) or high (***) registration quality. Tissue sections of reference and target images are 12 µm apart. (d-g) Bar charts showing the registration speed with different values of iterations (d), InitialRadius (e), Epsilon (f) or GrowthFactor (g) at ParamidLevels ranged L2-4. The registration quality is indicated by asterisks as in (c). Red outlines indicate default settings (in d, e-g). The same image set as used in (c) was used.

Figure S2. **Depiction of the spatial activation of MAPK and mTOR in GEMM PAC samples.** (a) Scatterplot showing areas (%) of p4EBP1, pAKT, pERK and pp38 in ASCs, PACs, or IACs. Data are presented as mean ± standard deviation (SD) of average marker expression in all tumours per histotype found within one animal, individual dots represent individual GEMMs. (b) Bar plot showing the spatial expression of p4EBP1 and pERK in GEMM PACs. Individual tumours are plotted, grouped per animal. Dashed black line indicates a 15% OL level. (c) Pie chart illustrating the classification of murine PACs according to MAPK/mTOR and Null spatial phenotypes.

Figure S3. **Overview of MAPK, AKT, mTOR and p38 activation and depiction of the spatial activation of MAPK and p38 pathways in clinical samples.** (a) Schematic summary of p4EBP1, pAKT, pERK and pp38 visual analyses in human ASC and PAC samples. Red bar and grey bars indicate presence or absence of expression of the indicated markers,

respectively. Replicate spots are marked by a dividing line in the bar. (b) Representative images illustrating p4EBP1, pAKT, pERK and pp38 expression in samples shown in (a). '++' indicates strong staining; '+' indicates weak staining; '-' indicates absence of expression. (c) Left: scatterplot depicting the areas (%) of pp38, pERK, pAKT, as well as pp38/pERK and pp38/pAKT OL in 13 individual clinical ASCs. Right: images of pp38 and pERK staining in eight $pp38^{positive}$ clinical ASCs. (d) Left: scatterplot depicting the areas (%) of pp38, pERK, and pp38/pERK overlap in 25 individual clinical PACs. Right: images of pp38 and pERK staining in 10 $pp38^{positive}$ clinical PACs. Scale bar, 500 µm.

Figure S4. Overview of *KRAS* mutation status of clinical samples and their distribution in the spatial signature classes. (a) Bar plot showing that 3 *KRAS* mutant and 2 *KRAS* wildtype ASC samples (5 of 13 ASCs in total) located to both MAPK/mTOR and Null signature classes. (b) Bar plot showing that within PACs of known *KRAS* status (15 out of 25), *KRAS* mutant samples (5 of 15) have a Null signature.

FIGURE S1

a

Spa-R optimizer configuration

GrowthFactor: 1.050000e+00
Epsilon: 1.500000e-06
InitialRadius: 1.8e-03
PyramidLevels = 3

b

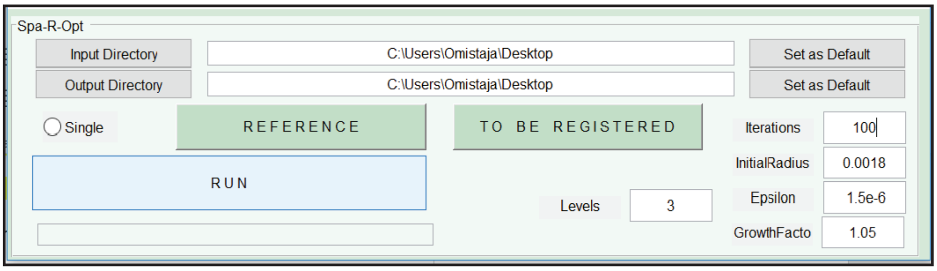

c

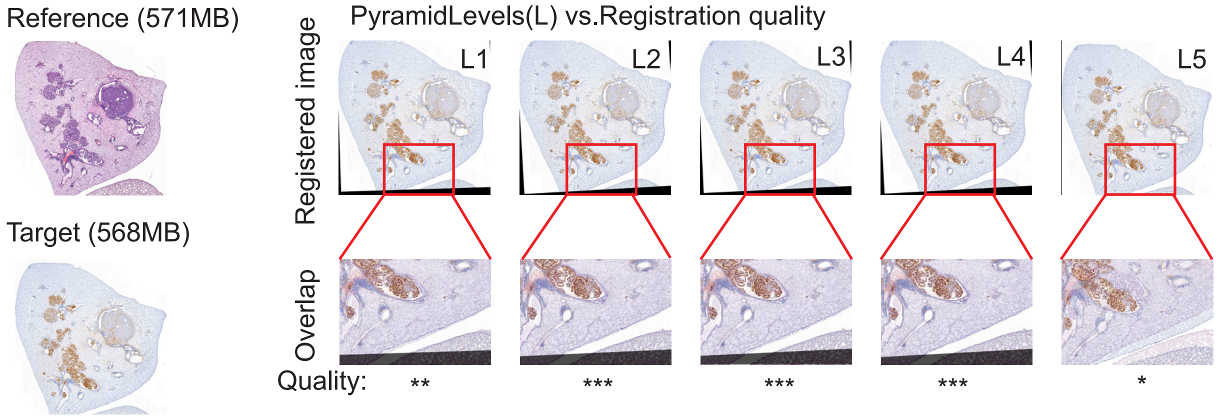

d

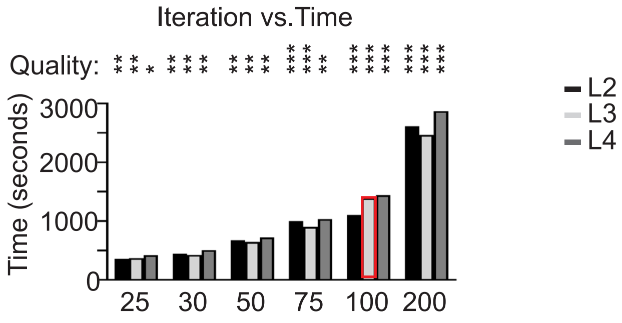

e

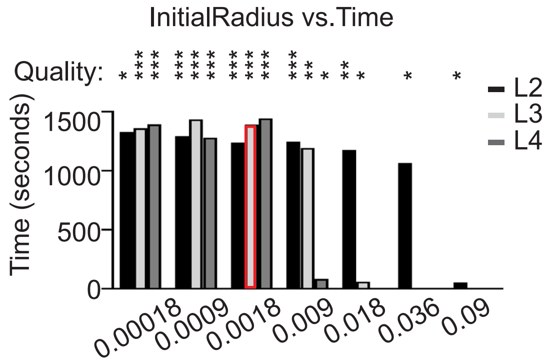

f

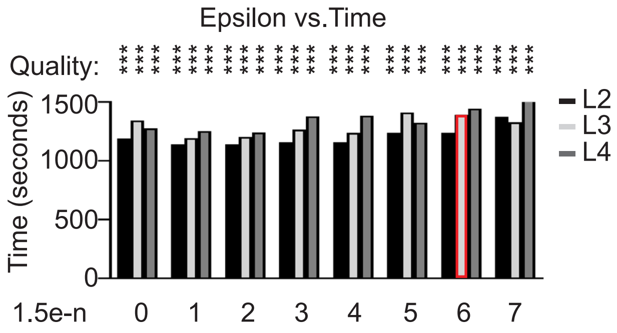

g

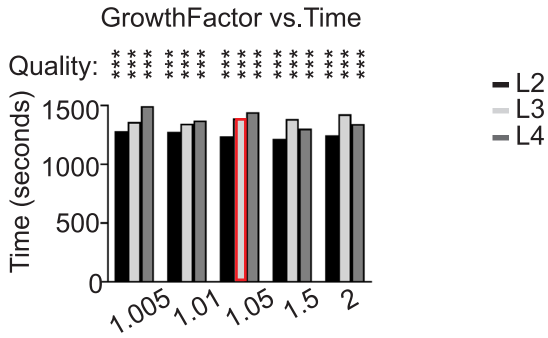

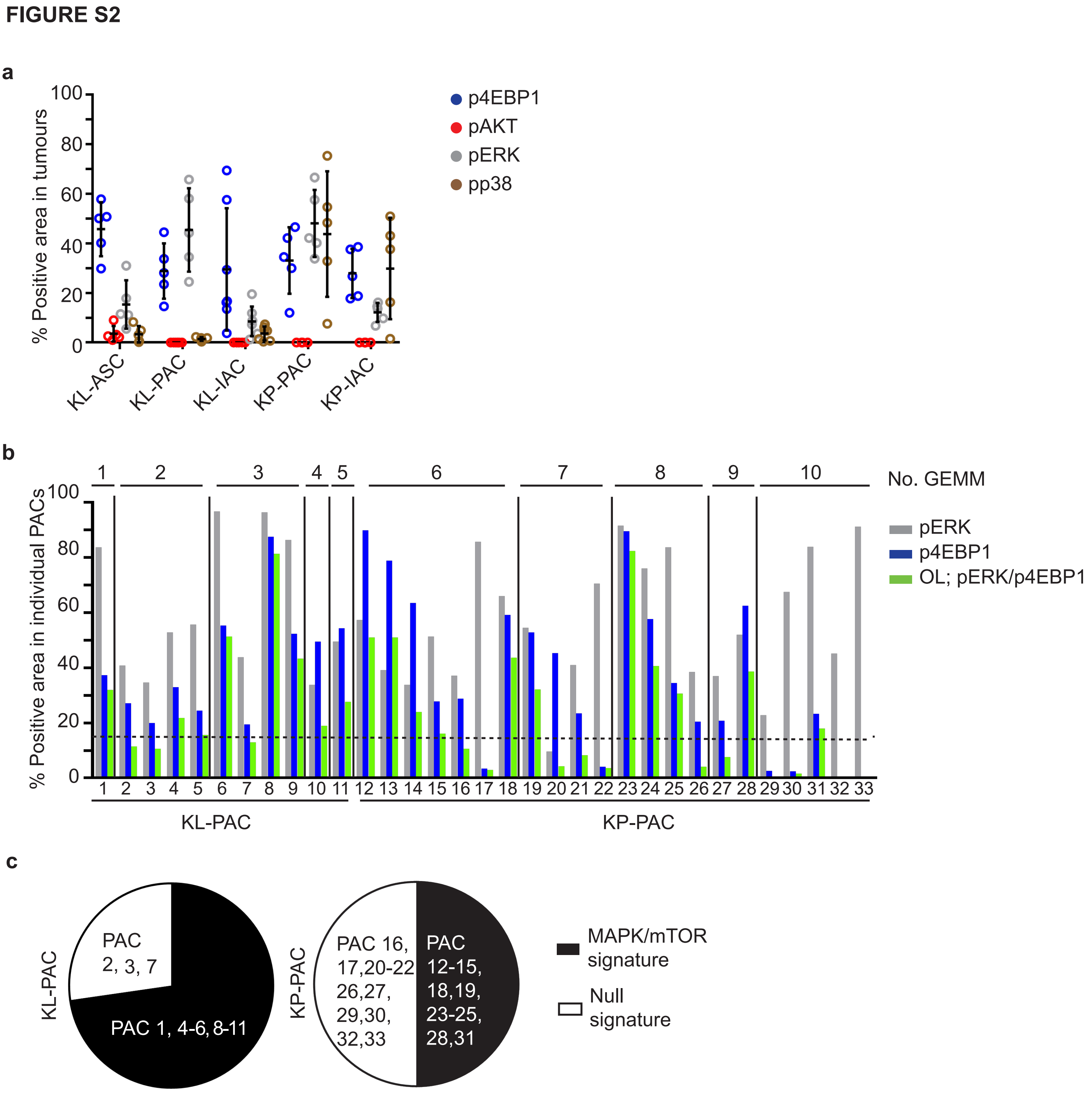
FIGURE S2
a
% Positive area in tumours
p4EBP1
pAKT
pERK
pp38
KL-ASC
KL-PAC
KL-IAC
KP-PAC
KP-IAC
b
No. GEMM
% Positive area in individual PACs
pERK
p4EBP1
OL; pERK/p4EBP1
KL-PAC
KP-PAC
c
KL-PAC
PAC 2, 3, 7
PAC 1, 4-6, 8-11
KP-PAC
PAC 16, 17,20-22 26,27, 29,30, 32,33
PAC 12-15, 18,19, 23-25, 28,31
MAPK/mTOR signature
Null signature

FIGURE S3

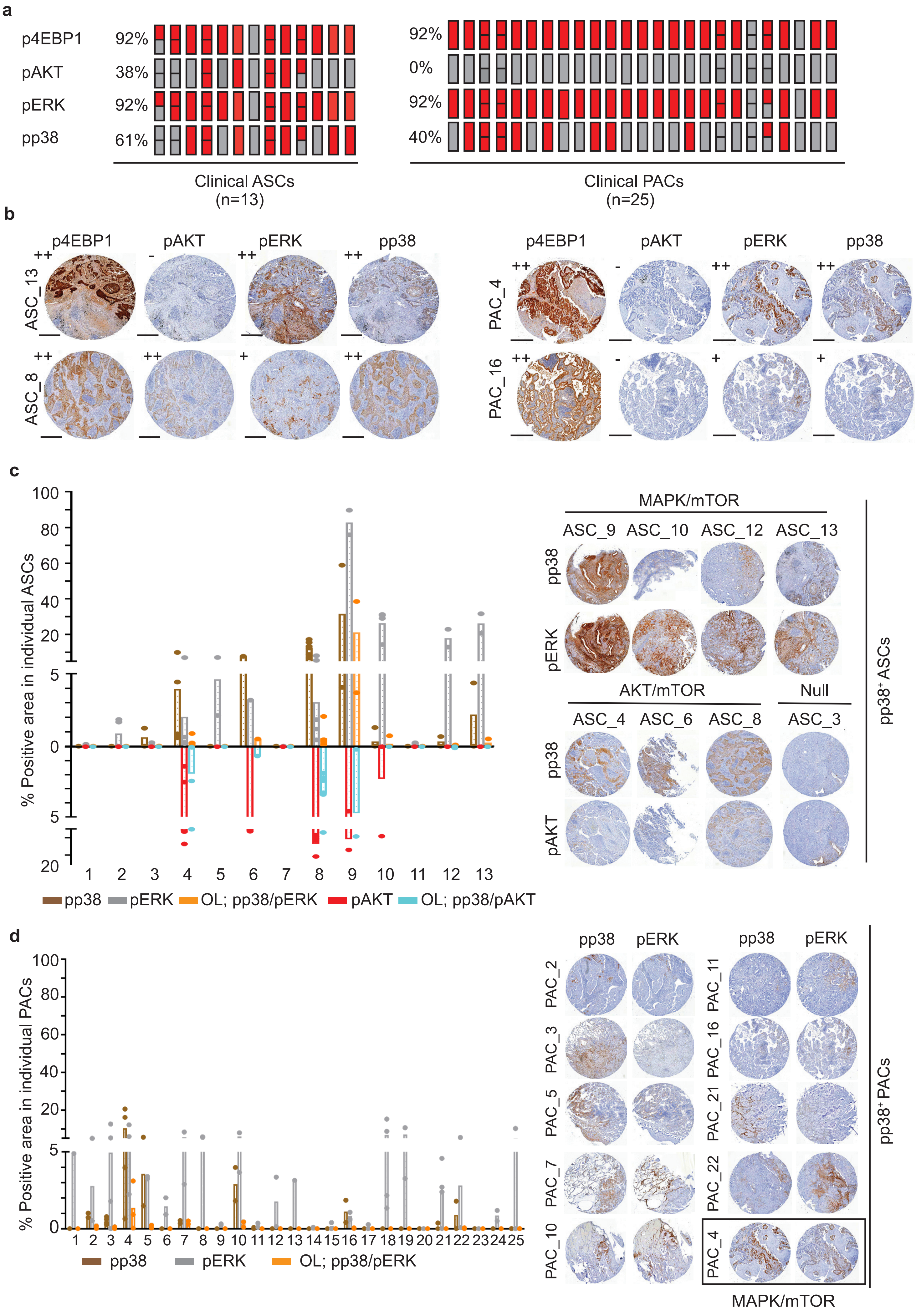

a
p4EBP1
pAKT
pERK
pp38
92%
38%
92%
61%
92%
0%
92%
40%
Clinical ASCs
(n=13)
Clinical PACs
(n=25)
b
p4EBP1
pAKT
pERK
pp38
ASC_13
ASC_8
PAC_4
PAC_16
c
% Positive area in individual ASCs
MAPK/mTOR
ASC_9
ASC_10
ASC_12
ASC_13
AKT/mTOR
ASC_4
ASC_6
ASC_8
Null
ASC_3
pp38⁺ ASCs
pp38
pERK
OL; pp38/pERK
pAKT
OL; pp38/pAKT
d
% Positive area in individual PACs
PAC_2
PAC_3
PAC_5
PAC_7
PAC_10
PAC_11
PAC_16
PAC_21
PAC_22
PAC_4
MAPK/mTOR
pp38⁺ PACs
pp38
pERK
OL; pp38/pERK

FIGURE S4

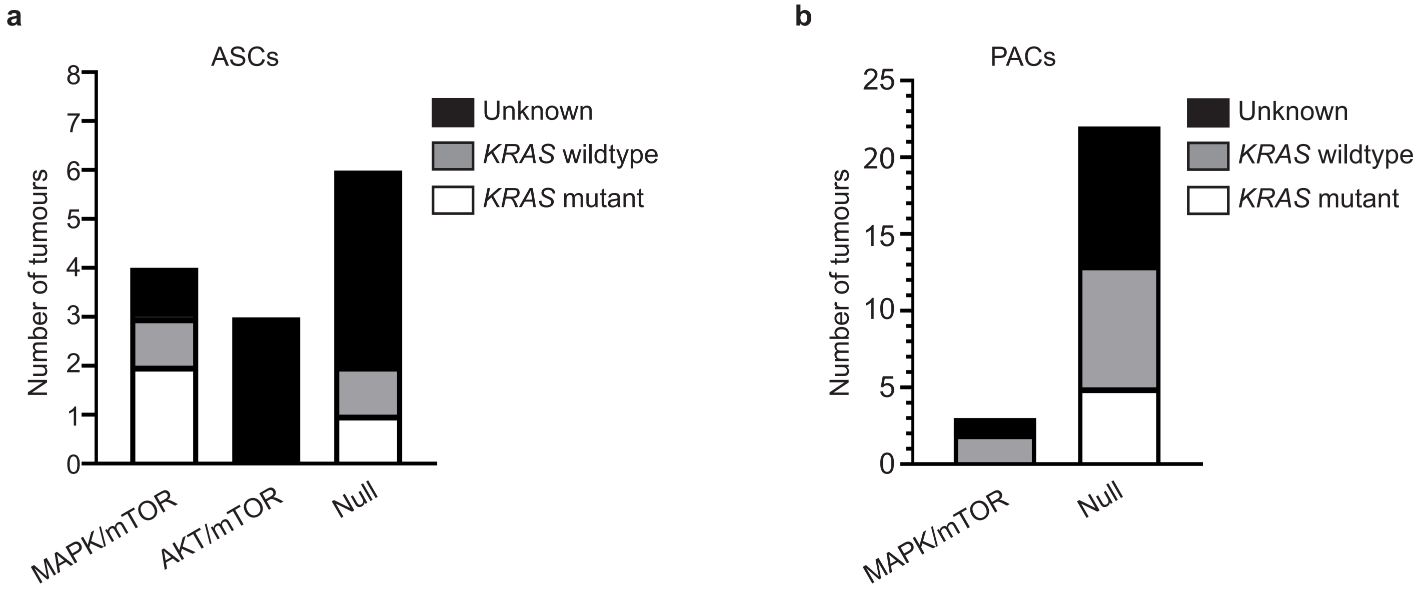
a
ASCs
Number of tumours
8
7
6
5
4
3
2
1
0
Unknown
KRAS wildtype
KRAS mutant
MAPK/mTOR
AKT/mTOR
Null
b
PACs
Number of tumours
25
20
15
10
5
0
Unknown
KRAS wildtype
KRAS mutant
MAPK/mTOR
Null
